# Morphologic and molecular evaluation of *Chlamydia trachomatis* growth in human endocervix reveals distinct growth patterns

**DOI:** 10.3389/fcimb.2014.00071

**Published:** 2014-06-10

**Authors:** Maria E. Lewis, Robert J. Belland, Yasser M. AbdelRahman, Wandy L. Beatty, Ashok A. Aiyar, Arnold H. Zea, Sheila J. Greene, Luis Marrero, Lyndsey R. Buckner, David J. Tate, Chris L. McGowin, Pamela A. Kozlowski, Michelle O'Brien, Rebecca A. Lillis, David H. Martin, Alison J. Quayle

**Affiliations:** ^1^Department of Microbiology, Immunology and Parasitology, Louisiana State University Health Sciences CenterNew Orleans, LA, USA; ^2^Department of Microbiology, Immunology and Biochemistry, University of Tennessee Health Sciences CenterMemphis, TN, USA; ^3^Department of Microbiology and Immunology, Faculty of Pharmacy, Cairo UniversityCairo, Egypt; ^4^Department of Molecular Microbiology, Washington University School of MedicineSt. Louis, MO, USA; ^5^Section of Infectious Diseases, Department of Medicine, Louisiana State University Health Sciences CenterNew Orleans, LA, USA

**Keywords:** bacterial persistence, *Chlamydia trachomatis*, endocervix, human, interferon gamma, indole

## Abstract

*In vitro* models of *Chlamydia trachomatis* growth have long been studied to predict growth *in vivo*. Alternative or persistent growth modes *in vitro* have been shown to occur under the influence of numerous stressors but have not been studied *in vivo*. Here, we report the development of methods for sampling human infections from the endocervix in a manner that permits a multifaceted analysis of the bacteria, host and the endocervical environment. Our approach permits evaluating total bacterial load, transcriptional patterns, morphology by immunofluorescence and electron microscopy, and levels of cytokines and nutrients in the infection microenvironment. By applying this approach to two pilot patients with disparate infections, we have determined that their contrasting growth patterns correlate with strikingly distinct transcriptional biomarkers, and are associated with differences in local levels of IFNγ. Our multifaceted approach will be useful to dissect infections in the human host and be useful in identifying patients at risk for chronic disease. Importantly, the molecular and morphological analyses described here indicate that persistent growth forms can be isolated from the human endocervix when the infection microenvironment resembles the *in vitro* model of IFNγ-induced persistence.

## Introduction

*Chlamydia trachomatis* is an obligate intracellular bacterium and serovars D-K are tropic for the columnar and transitional epithelial cells of the genital tract. Chlamydial infections in women are generally asymptomatic and therefore often go undetected and untreated (Brunham and Rey-Ladino, 2005). Natural history studies indicate untreated infections can be asymptomatic for substantial periods of time, can spontaneously resolve or can progress to cause complications (Parks et al., 1997; Golden et al., 2000; Joyner et al., 2002; Morre et al., 2002; Molano et al., 2005; Geisler et al., 2008). Infection most commonly occurs in the endocervix and can result in cervicitis. If bacteria ascend into the endometrium and Fallopian tubes chronic infection can lead to pelvic inflammatory disease (PID). Approximately 11% of women with PID will subsequently develop tubal factor infertility, but, as many of these infections are also clinically silent, they also remain undiscovered until reproductive consequences ensue (Cohen and Brunham, 1999).

Why so many chlamydial infections are so extended in their duration is not well understood, but does indicate the organism is capable of adapting to, or evading, specific immune and environmental conditions (Brunham and Rey-Ladino, 2005). One strategy documented *in vitro* for immune evasion or adaptation in the human host is the ability of *C. trachomatis* to enter into a persistent growth form (Beatty et al., 1994b; Belland et al., 2003a). This bacterial form is viable but non-cultivable and results in an extended relationship between the pathogen and its host cell (*ibid*). Compelling, but thus far indirect evidence, for this alternative mode of growth *in vivo* includes documentation of recurrent disease when re-infection is unlikely, and the detection of chlamydial antigen or nucleic acid in the absence of cultivability (Nagasaki, 1987; Patton et al., 1994; Dean et al., 2000).

Classic *in vitro* studies have shown *C. trachomatis* has a unique developmental cycle that normally alternates between an infectious elementary body (EB) and a non-infectious reticulate body (RB) (Abdelrahman and Belland, 2005). EBs attach to, and invade, susceptible cells where they are internalized in membrane bound vacuoles termed inclusions (*ibid*). EBs, now known to possess some metabolic activity (Omsland et al., 2012), then differentiate into highly metabolically active RBs, undergo repeated cycles of binary fission and then differentiate back to EBs whence they are released from the host cell by lysis or extrusion to infect neighboring cells (Abdelrahman and Belland, 2005; Hybiske and Stephens, 2007). The entire developmental cycle may take 30–48 h, dependent on the serovar, with temporally distinct patterns of gene expression categorized as early, mid-cycle and late that correlate with *C. trachomatis* growth stages (Belland et al., 2003b). Stressful growth conditions that are also likely to be encountered *in vivo* can induce an alternate, persistent growth mode *in vitro* (Wyrick, 2010). These stressors include, nutrient and iron deprivation (Raulston, 1997; Igietseme et al., 1998), specific antibiotics (Matsumoto and Manire, 1970; Clark et al., 1982), co-infection with herpes simplex virus (HSV) (Vanover et al., 2008), exposure of infected cells to the danger signal adenosine (Pettengill et al., 2009), and interferon gamma (IFNγ) (Beatty et al., 1993), the latter of which, under optimal conditions, is believed to be a key immune mediator in resolution of, and subsequent protection from, infection(Rank and Whittum-Hudson, 2010; Aiyar et al., 2014). Persistent bacterial forms, induced by IFNγ, are morphologically characterized as large, atypical, or aberrant RBs in which binary fission appears to be arrested (Byrne et al., 1986; Beatty et al., 1993, 1994b; Wyrick, 2010). Molecularly, gene expression profiles associated with persistent forms are consistent with RBs blocked in binary fission and arrest of the developmental cycle at the stage just preceding late gene expression (Belland et al., 2003a). Removal of IFNγ generally reverses these changes such that aberrant RB re-enter the developmental cycle and differentiate into infectious EBs. Well-characterized *in vitro* models indicate that IFNγ acts against *C. trachomatis* via nutrient deprivation (Beatty et al., 1993). Specifically, IFNγ induces the tryptophan-catabolizing enzyme, indoleamine 2,3-dioxygenase (IDO1), thereby depriving *C. trachomatis*, a tryptophan auxotroph, of this essential amino-acid (Byrne et al., 1986; Taylor and Feng, 1991; Beatty et al., 1994a,b). Depending on the concentration and duration of exposure to IFNγ, and consequently the environmental tryptophan levels, *C. trachomatis* either enters into a persistent state of growth, or can be eradicated (Byrne et al., 1989). Importantly, genital serovars of *C. trachomatis* can uniquely synthesize tryptophan through indole salvage (Fehlner-Gardiner et al., 2002), suggesting that exogenous sources of indole, likely microbial-derived in the natural environment, may extend or permit the survival of *C. trachomatis* in the presence of IFNγ (Fehlner-Gardiner et al., 2002; Caldwell et al., 2003; Aiyar et al., 2014).

While *in vitro* models have proven very insightful in elucidating chlamydial growth modes under highly controlled conditions, there have been no definitive studies that directly establish whether persistent growth forms as described above are an *in vivo* survival mechanism for *C. trachomatis* (Wyrick, 2010). In fact, there is a paucity of information describing *C. trachomatis* growth in the human genital tract milieu, the composition of this milieu, how endogenous and exogenous co-factors alter the composition, and the resultant effects on *C. trachomatis*. Variability in these co-factors may be critical in determining whether *C. trachomatis* survives or is eradicated by host immune responses. Addressing this gap in our knowledge will likely reveal the mechanisms by which *C. trachomatis* maintains *in vivo* reservoirs of infection. It will also provide crucial normative data to aid in the design of diagnostics, vaccines, and adjunct therapies that could classify, target, and eliminate human *C. trachomatis* infections. Therefore, the objective of the study described here was to develop methodology to harvest, preserve, and analyze cells and secretions from the human endocervix that would permit parallel molecular and morphological analyses of *C. trachomatis*, along with analyses of the immune and environmental milieu in which it survives. Using this novel multifaceted approach, we were able to identify highly contrasting patterns of bacterial growth, and associated cervicovaginal environment factors, in two pilot patients. These preliminary results indicate that this approach may pave the way to the establishment of a biomarker panel suitable for assessing chlamydial growth patterns and disease outcomes of *C. trachomatis* infections in women. Importantly, the contrasting molecular and morphological characteristics observed in these two patients provide the first evidence for the existence of persistent growth forms in the human genital tract.

## Materials and methods

### Study population and clinic procedures

Institutional Review Board approval for this study was obtained from LSU Health Sciences Center. Women aged 18–28 years and attending the Delgado STD Clinic were asked to participate and then enrolled in this study if they had a high probability of chlamydial infection based on the following criteria: a recent positive NAAT screening test for *C. trachomatis*; recent sexual contact with a male suspected of chlamydial infection; or clinical evidence of cervicitis. Exclusion criteria were as follows: pregnancy; underlying chronic disease; use of steroids or antibiotics within the last 2 weeks; sexual intercourse within the last 12 h; current menstrual bleeding; documented infection with human immunodeficiency virus; or a history of genital herpes. Samples were excluded from the analyses if women were *C. trachomatis* NAAT or culture negative at the enrollment visit. All *Chlamydia trachomatis*-infected women were treated with azithromycin at this treatment/enrollment visit. Two groups of women were sequentially enrolled into the study; variations in the cytobrush and/or endocervical culture swab collection fluid or processing was the only difference in the two groups.

### Sample collection

Pelvic samples were taken in the following order: (i) vaginal swab for a wet mount and a Gram stain preparation; (ii) vaginal swab placed in an InPouch (BioMed Diagnostics Inc.) for *Trichomonas vaginalis* culture; (iii) two sequential cervical cytobrush samplings, each one as a gentle 360° sweep of the cervical os, immersed in 1.5 ml collection fluid; (iv) endocervical swab immersed in endocervical transport medium (Ficarra et al., 2008) in a one dram vial with glass beads for *C. trachomatis* culture and genotyping; and (v) an endocervical swab for NAAT testing (*ibid*). In study 1 (the inclusion identification study), cytobrushes were immediately placed in a commercial transport fluid for liquid-based Papanicalou (PAP) testing (SurePath by BD Diagnostics or CytoLyt by Hologic). In study 2 (the ultrastructure and growth biomarker study), three changes were made to the protocol as follows: (1) cytobrushes were immediately immersed in a modified Electron Microscopy (EM) buffer (4% paraformeldehyde-1% glutaraldehyde); (2) the endocervical swab in endocervical transport medium from patients in study 2 was immediately vortexed in clinic and 25% of the sample was removed and placed in an equal volume of MasterPure tissue and cell lysis solution (Epicentre, Illumina); and (3) Merocel ophthalmic sponges (Medtronic Xomed Inc.) were placed in the posterior fornix of the vagina for 2 min to absorb secretions (Kozlowski et al., 2000). Sponges were immediately placed in cryovials and transported to the laboratory on ice, after which they were stored at −80°C until vaginal fluid was extracted.

### STD diagnostics

Endocervical swab specimens were used to determine the presence of *C. trachomatis* and *N. gonorrhoeae* using the Aptima Combo 2 test as instructed by the manufacturer (Genprobe). A vaginal wet preparation was made in the clinic, and bacterial vaginosis (BV) was later diagnosed by Gram stain, with a Nugent's score of ≥7 being positive (Nugent et al., 1991). InPouch culture for *T. vaginalis* screening was read at baseline, 48 and 72 h (Ficarra et al., 2008). Swab specimens in endocervical medium were immediately frozen until they were processed for semi-quantitative culture for inclusion forming units (IFU) and for genotyping of *C. trachomatis*, as previously described (*ibid*).

### Cytobrush processing

Cytobrushes from study 1 were centrifuged, resuspended in 500 μl PBS, and concentrated endocervical cell preparations were dropped into PAP-pen circumferenced circles on glass slides, dried, fixed in 90% methanol for 10 min and stored at −20°C until immunofluoresecent analyses (IFA) were performed. Cytobrushes in modified EM buffer were vortexed, washed twice, and resuspended in PBS, then cells were enumerated. Twenty percent of the sample was used to make slides as described above, and the remainder was further processed for EM analysis if inclusions were noted on an IFA screen.

### Immunofluorescent staining, counting of inclusions, and deconvolution microscopy

Cells on slides were rehydrated in PBS and incubated overnight at 4°C with a blocking agent (Background Sniper, Biocare Medical). Between 50 and 100% of each sample was used to count inclusions in the inclusion identification study, depending on the number of cells retrieved from the patient, and 100% was used in the EM/Biomarker study. An anti-chlamydial LPS antibody conjugated to fluorescein isothiocyanate (FITC) and which contains Evans Blue as a counterstain (Merifluor, Meridian) was used to visualize *C. trachomatis* forms; 4',6 diamidino-2-phenylindole (DAPI) was also applied to visualize nucleic acid (Molecular Probes). Slides were cover-slipped with Prolong Gold antifade reagent (Invitrogen) prior to the examination of inclusions. In samples with larger numbers of cells, we also investigated the expression of the chlamydial proteins OmcA and CT223. In brief, following rehydration and blocking, samples were incubated with Image-iT R FX Signal Enhancer followed by a mouse monoclonal antibody to OmcA (1:750, B12K, a kind gift of Dr. Li Shen) (Zhang et al., 1987) or a rabbit polyclonal anti-CT223 at (1:500, CT223 186, a kind gift of Dr. Dan Rockey) (Alzhanov et al., 2009). Samples were incubated with a secondary antibody (Alexa568 conjugated anti-mouse antibody or Alexa 594 conjugated anti-rabbit antibody, 1:500, Molecular Probes) followed by an anti-chlamydial LPS-FITC antibody (1:20, YVS 1683, Accurate). Dual stained slides were finally counterstained and cover-slipped as described above. All images were obtained with a Leica DMRXA automated upright epifluorescent microscope (Leica Microsystems); a Sensicam QE CCD (Cooke Corporation); and filter sets optimized for Alexa 488 (exciter HQ480/20, dichroic Q495LP, and emitter HQ510/20m), Alexa 568/594 (exciter HQ560/55x, dichroic Q595LP, and emitter HQ645/75m) and DAPI (exciter 360/40x, dichroic 400DCLP, and emitter GG420LP). Images were captured with a 63X objective (NA = 1.42) and a 2× zoom. Z-axis plane capture, deconvolution, and analysis were performed with Slidebook™ Deconvolution Software (Intelligent Imaging Innovations, Denver, CO).

### Ultrastructural analyses

Cervical cell samples were post-fixed in 1% osmium tetroxide (Polysciences Inc.) for 1 h as previously described (Belland et al., 2003a). Samples were then rinsed extensively in deionized water (dH_2_0) prior to en bloc staining with 1% aqueous uranyl acetate (Ted Pella Inc.) for 1 h. Following several rinses in dH_2_0, samples were dehydrated in a graded series of ethanol and embedded in Eponate 12 resin (Ted Pella Inc.). Thick sections (200–300 nm) obtained with a Leica Ultracut UCT ultramicrotome were stained with toluidine blue for initial screening of tissue for inclusions at the light microscopy level. Ultrathin sections of 95 nm were stained with uranyl acetate and lead citrate for detailed ultrastructural analyses of bacteria and inclusions on a JEOL 1200 EX transmission electron microscope (JEOL USA Inc.). The bacterial forms in identified inclusions were subsequently evaluated using the scientific image processing and analysis program, Fiji (Schindelin et al., 2012), as described here. After appropriately setting the scale, the freehand selection tool was used to create a rough outline of each inclusion. Image thresholding was used to remove background pixel density within the inclusion. The particle analysis function was used to enumerate the number of EBs per inclusion. Particles that were greater than 0.05 μm^2^ with a circularity between 0.45 and 1.0 were enumerated. These parameters identified immature EBs with an electron-dense core, electron dense mature EBs, circular RBs, RBs undergoing binary fission, and atypical RBs that were larger in size and displayed multiple septum formation. For those inclusions in which the background density did not permit a single threshold to be applied, an alternative procedure was used. Image thresholding was applied to the outlined inclusions to reveal immature and mature EBs that were sized for their areas. The following procedure was used to enumerate and size the RBs and atypical RBs. First, the thresholded image containing mature/immature EBs was used to create a mask image in which all the EBs were outlined. This mask was subtracted from the original micrograph to remove all EBs. A new image threshold was now applied to subtract the background and enumerate and size RBs and atypical RBs.

### qPCR for *C. trachomatis* genes and genome copy number quantitation

Endocervical swab samples in MasterPure lysis solution (Epicenter, Illumina) were processed for total nucleic acid according to the manufacturers instructions and stored at −80°C until use. For each patient, 30% of each sample was used to determine *C. trachomatis* genome copy number. RNA was removed by digestion with RNaseA, the DNA was re-purified by isopropanol precipitation and samples (150 ng/well) were then analyzed by Taqman™ qPCR in triplicate, including a no-template control, using an Applied Biosystems PRISM 7700 Sequence Detection System. The remaining total nucleic acid was treated with DNase and re-precipitated. Each of the samples was subjected to qRT-PCR using Taqman primer/probe sets specific for the mRNAs encoded by the genes *euo* and *omcB*. Equivalent amounts of RNA (20 ng) were used to measure expression levels in triplicate and a no-RT control was included for each primer/probe set. Normalization was performed using *C. trachomatis* genome copy numbers, as previously described (Ibana et al., 2011).

### Detection of IFNγ and indoles in vaginal secretions

Vaginal fluid was eluted from Merocel sponges in a spin assembly apparatus, and secretion volumes and dilution factors were calculated as previously described for Weck-Cel sponges (Kozlowski et al., 2000). Total protein was assayed using a Pierce BCA protein assay kit (Thermo Fisher Scientific). IFNγ was quantified by a cytometric bead array assay (MILLIPLEX MAP Immunology Multiplex Assay). Millipore, Billerica, MA) as previously described (Buckner et al., 2011, 2013). Total indoles were quantified using Salkowski's test (Salkowski, 1885), modified as described by Szkop et al. (2012). In brief, an equimolar mixture of multiple indoles (Sigma-Aldrich, Inc., St. Louis, MO, USA) was used to generate a standard curve by measuring absorbance at 530 nm after incubation with Salkowski's reagent. Indoles that can be detected by this test include indole, 3-methyl indole, indolic acids, and indolic alcohols. Samples were processed in parallel, following which the standard curve was used to determine the total concentration of indoles in the sample. Concentrations were corrected for the sample dilution factor.

## Results

### Study population and characteristics of endocervical infection

Seventy-five women were recruited into the study and samples from 37 women were included in the final analyses; 29 women were excluded because they were *C. trachomatis* NAAT and/or culture negative, and 9 were excluded because samples were insufficient or could not be processed in a timely fashion. The median age of the women with samples included in the study was 22 years (range 18–30 years). Fourteen of the women had mucopurulent cervicitis (MPC) (38%). Semi-quantitative culture of *C. trachomatis* indicated infectious burden in the endocervix varied considerably, with IFU per endocervical swab ranging from 7 to 336,168 IFU (median 4802). None of the women were positive for *Neisseria gonorrhoeae*, but 3 (8.1%) were co-infected with *T. vaginalis*. Twenty-one women (56.8%) were diagnosed with BV (Nugent score 7–10) and 7 (18.9%) had an intermediate (4–6) Nugent score (Nugent et al., 1991).

### Identification of intact chlamydial inclusions in epithelial cells harvested from cervical cytobrush specimens

**Figure 1:**
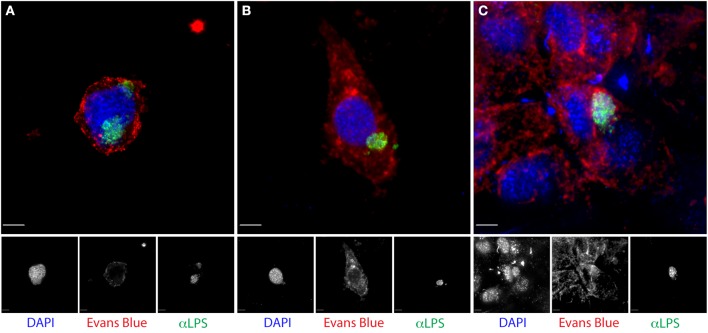
**Identification of chlamydial inclusions in endocervical cells retrieved by cytobrush from *C. trachomatis-*infected women**. Cytobrush specimens immediately placed in **(A)** Surepath or **(B,C)** Cytolite were processed as described in the methods, stained with anti-chlamydial LPS-FITC (green), Evans blue (red) and DAPI (blue) and visualized by fluorescent deconvolution microscopy. Note the morphology and staining pattern in **(A)** suggests a single inclusion that is wrapped around the nucleus. Scale bar is 5 μm.

**Figure 2:**
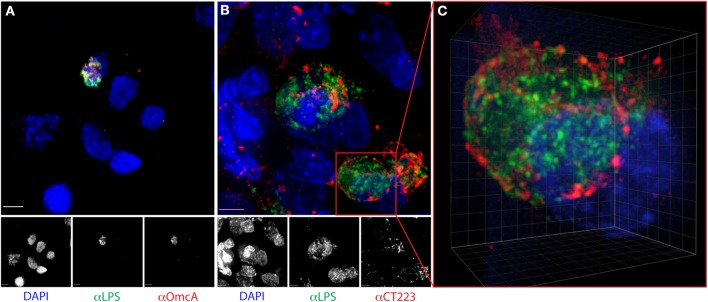
**Distribution of chlamydial LPS, OmcA, and CT223 in *C. trachomatis-*infected epithelial cells harvested from human endocervix**. Cervical cells were dual-labeled with anti-chlamydial LPS (green) and **(A)** OmcA (red) or **(B)** CT223 (red), counterstained with DAPI (blue) and visualized by fluorescence deconvolution microscopy, as described in the methods. Colocalization of antigen is visualized in yellow. Scale bar is 5 μm. Image **(C)** is a 3-dimensional maximum intensity projection extracted from a deconvolved stack of an infected cell in image **(B)**. Note the irregular “dashed line” membranous staining of CT223. Grid is 1 μm.

We previously described the utility of cytobrushes for the non-invasive and longitudinal retrieval of cervical lymphocytes (Ficarra et al., 2008). Here, we established that cytobrush sampling can be used to harvest and enumerate *C. trachomatis-*infected cervical epithelial cells. The original protocol was modified such that cytobrushes were immersed immediately upon collection into liquid-based Pap test collection fluid. Bright-field microscopy revealed epithelial cells in these samples retained their characteristic morphology, permitting the detection of chlamydial antigen and nucleic acid by fluorescence. DAPI staining for DNA and immunofluorescent staining for chlamydial LPS detected inclusions in 79.2% of samples (Figures 1A–C). The median number of cells with inclusions in positive samples was 11 (range 2–50), with no correlation observed between inclusion number and IFU (*R*^2^ = 0.08). Clusters of multiple infected cells were often observed, and neutrophils frequently appeared to be associated with the infected cells (Figure 2B). However, we note that samples always included infected and uninfected “bystander” cells (Figures 1C, 2B). In samples that had larger numbers of cells, dual staining was performed using antibodies recognizing chlamydial LPS and OmcA, a late cycle-expressed protein, or chlamydial LPS with CT223, an inclusion membrane protein. We observed that our results paralleled *in vitro* studies, with only EB-size forms staining for OmcA (Figure 2A) and CT223 irregularly distributed in a patchy “dash-like” distribution at the inclusion surface (Figures 2B,C) (Alzhanov et al., 2009).

### Ultrastructural analyses of cervical inclusions provide evidence for aberrant forms

The finding that immunofluorescence could be used to screen for inclusion frequency in cervical samples encouraged us to next develop a protocol that permitted a detailed ultrastructural and molecular examination of these bacterial forms, along with molecular studies of the cervicovaginal milieu. There were three modifications of the sample collection protocol for the 13 women enrolled into this component of the study. First, cytobrushes were collected directly into a modified EM fixative; the formulation of this fixative maintained the antigenic integrity of chlamydial LPS and allowed visualization by standard IFA in samples processed within 6 h of collection. This enabled us to screen samples by IFA prior to processing for EM. Second, 25% of the endocervical swab sample was immediately stabilized in MasterPure buffer in the clinic. This enabled parallel analysis of chlamydial DNA and RNA from the same sample used for determination of the IFU count. Third, vaginal fluid was collected using an ophthalmic sponge for quantification of IFNγ and indoles in these secretions.

**Figure 3:**
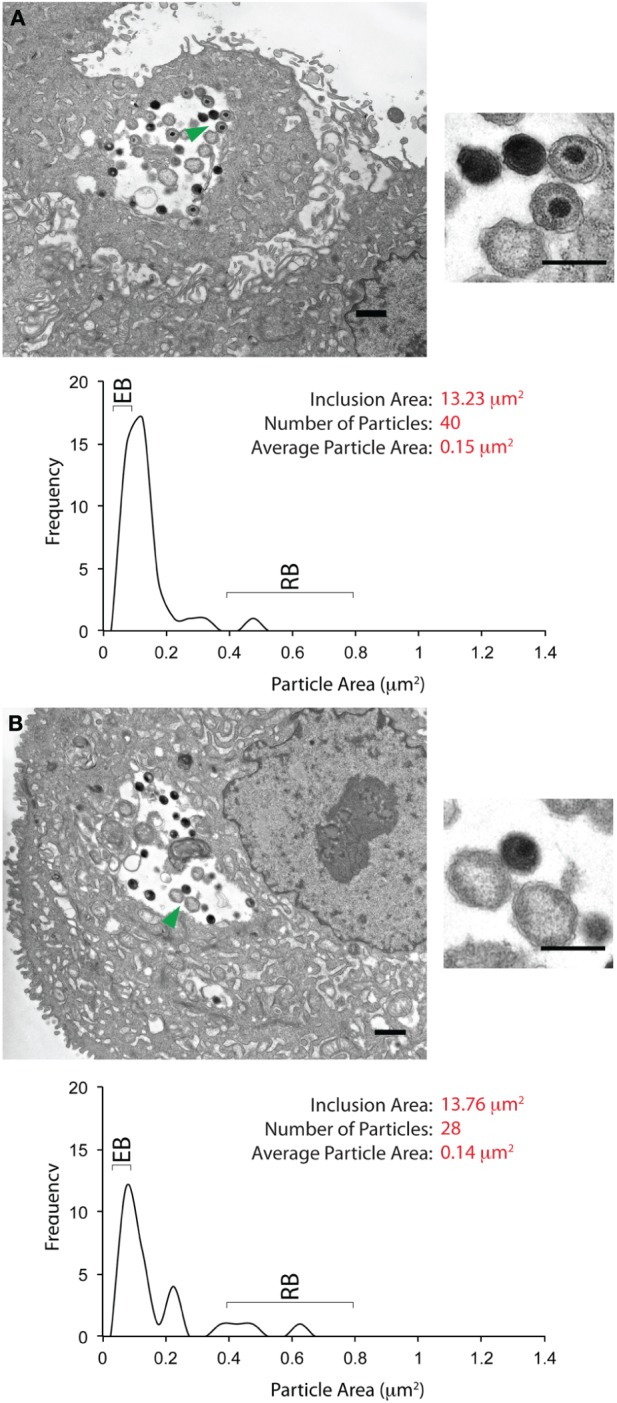
**Ultrastructural images of inclusions from the cervix of a single *C. trachomatis* patient. (A)** An inclusion-containing electron micrograph from Patient 1, processed and analyzed as described in the Materials and Methods section. The entire micrograph is shown in the large image on the left, with the scale bar indicating a distance of 1 μm. The smaller image on the right is a magnified view of the features indicated by the green arrow. For this image, the scale bar indicates a distance of 0.5 μm. Mature and immature EBs can be observed in the magnified view. Quantification of the inclusion and its contents is indicated below the image. The inclusion area was 13.23 μm^2^. Forty EBs, RBs, and atypical RBs were counted as particles within the inclusion, with an average particle area of 0.14 μm^2^. The plot indicates the frequency distribution of particles sized by their cross-sectional areas. The anticipated cross-sectional areas for EBs and RBs are indicated in the frequency plot. The plot indicates the frequency distribution of particles sized by their areas. **(B)** A second inclusion-containing electron micrograph from Patient 1, processed and analyzed as described above. The smaller image on the right indicates a magnified view of the area highlighted by the green arrowhead on the larger image. Two EBs and two RBs can be observed in the magnified view. Quantification of inclusion area, particle area, and particle area frequency distribution indicates the characteristics of this inclusion to be very similar to those in **(A)**.

A fraction (20%) of each cytobrush sample was screened for the presence of inclusions by immunofluorescence using an anti-chlamydial LPS antibody. Inclusions were detected by this method in 5 of 13 samples, two of which had multiple (>10) inclusions and were hence selected for further analyses. The remaining 80% of the cytobrush for each of these two samples were processed for EM and scanned for inclusions. Using this protocol, we were able to identify and examine three inclusions in Patient 1 and five inclusions in Patient 2. There was a striking difference in the morphology of bacterial forms in the inclusions from these two subjects. Two EM images from Patient 1 are shown in Figures 3A,B. The morphological characteristics in the third inclusion were similar to these two inclusions. Each image displays the inclusion cross-sectional area, within regions indicated by the green arrowheads magnified. A graph indicating the distribution of particle sizes (as area) is also shown. Inclusions from Patient 1 had an area of approximately 13–14 μm^2^, with 25–40 “particles” within them. The average area for each particle was approximately 0.15 μm^2^. Particles were sized by area rather than diameter because some of them were shaped irregularly. The area of an EB (diameter 0.2–0.3 μm) is anticipated to be in 0.07–0.12 μm^2^. The area within an RB (diameter 0.7–1 μm) is expected to be about 0.4–0.8 μm^2^ (Ward, 1983; Wyrick, 2010). Particle analyses indicated that the particles within inclusions from Patient 1 were skewed toward the area of an EB. The morphology of the particles within these inclusions is indicated in the particles highlighted by the green arrowheads. Electron dense EBs, immature EBs, and RBs were observed.

**Figure 4:**
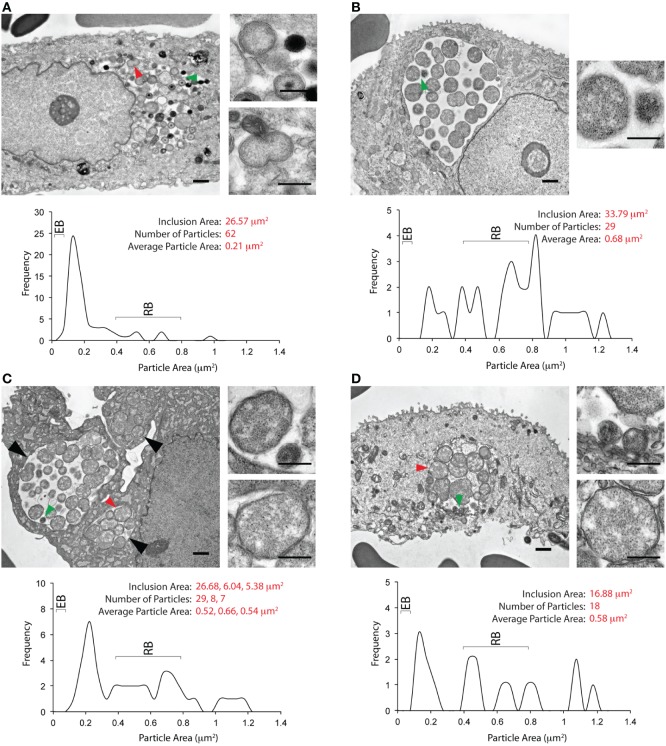
**Ultrastructural images from the cervix of a second *C. trachomatis* infected patient**. **(A)** An inclusion-containing electron micrograph from Patient 2, processed and analyzed as described for Figure 3. The entire micrograph is shown in the large image on the left, with the scale bar indicating a distance of 1 μm. The smaller images on the right are magnified views of the features indicated by the green (upper image) and red (lower image) arrows. For these images, the scale bar indicates a distance of 0.5 μm. The area indicated by the green arrowhead contains an EB, and an RB. The area indicated by the red arrowhead contains an RB undergoing binary fission. Quantification of the inclusion and its contents is shown below the images. The inclusion area was 26.57 μm^2^. Sixty-two EBs, RBs, and atypical RBs were counted as particles within the inclusion, with an average particle area of 0.21 μm^2^. The plot indicates the frequency distribution of particles sized by their cross-sectional areas. The anticipated cross-sectional areas for EBs and RBs are indicated in the frequency plot. **(B)** A second inclusion-containing electron micrograph from Patient 2. The smaller image on the right indicates a magnified view of the area highlighted by the green arrowhead on the larger image. A RB and an EB-sized particle can be observed in the magnified view. Quantification of inclusion area, particle area, and particle area frequency distribution indicates the characteristics of this inclusion to differ significantly the inclusion shown in **(A)** or Figure 3. Very few particles with the area of an EB were observed. The average area, 0.68 μm^2^, was skewed toward the anticipated area of a RB, with several larger particles also observed. **(C)** A third inclusion-containing electron micrograph from Patient 2. Three inclusions were observed in this micrograph, indicated by the large black arrows, at least two of which are within the same cell. The largest inclusion contained particles with the characteristic area of RBs and EBs, as highlighted by the green arrow, and displayed in the upper image on the right. The two smaller images contained particles with areas corresponding to those of RBs, as highlighted by the red arrow, and displayed in the lower image on the right. Quantification of the three inclusions is indicated below. The largest inclusion had an area of 26.68 μm^2^, whereas the smaller inclusions had areas of 5–6 μm^2^. The average area of particles within these three inclusions was similar to that observed in **(B)**, and distinct from the observations in **(A)**. **(D)** A fourth inclusion-containing micrograph from Patient 2. The characteristics of this inclusion are close to those observed for the inclusions in **(B,C)**. The green arrowhead indicates particles with the area of EBs (upper magnified image on the right), while the red arrowhead indicates a particle with the area of an RB (lower magnified image on the right). Similar to the inclusions seen in **(B,C)**, the particles within this inclusion were skewed toward the size of RBs, with an average area of 0.58 μm^2^.

EM images from five unique inclusions were obtained from Patient 2. The entire inclusion cross-sectional area was not obtained for the first inclusion by EM, preventing calculation of all parameters used for the other inclusions. EB morphotypes predominated in this inclusion (Supplementary Figure 1). The second inclusion is shown in Figure 4A. The inclusion membrane was difficult to visualize for this inclusion; therefore inclusion area was calculated by subtracting a mask created using cytoplasmic/nuclear pixel density. The area of this inclusion, 26 mm^2^, was larger than the areas for inclusions from Patient 1. Particles within this inclusion displayed the morphological characteristics of EBs, immature EBs, and RBs. Some atypical/abnormal RBs (ABs) displaying unequal binary fission were also observed. The average particle area within this inclusion was 0.21 μm^2^, reflecting a larger number of RBs than the inclusions shown in Figure 3. The other three inclusions from this patient (Figures 4B–D) displayed similar characteristics to each other. They contained a large number of RBs/ABs, with very few EBs, reflected in an average particle area of 0.68 μm^2^. The green arrowhead in Figure 4B indicates particles with RB and EB morphology. While many RBs displayed equal binary fission, some abnormal binary fission events were also observed. The micrograph shown in Figure 4C contains three inclusions indicated by the large black arrowheads, two of which are within the same cell. The third inclusion may also be within that cell. While inclusion areas for the first two inclusions were determined by automatic thresholding, the area for the third inclusion was determined by manually outlining the inclusion. The largest inclusion contained EBs, RBs, and several ABs, as evaluated by particle area. No EB sized particles were observed in the two smaller inclusions. The average area for particles within these three inclusions was skewed toward RBs. As classified by area, several atypical RBs were observed in all three inclusions. The fifth inclusion from this patient is shown in Figure 4D. The morphological characteristics of this inclusion resemble those observed for the inclusions shown in Figures 4B,C. This inclusion contained a few EB-sized particles (green arrowhead), although these particles did not have the electron density of mature EBs, or the electron dense core observed in immature EBs. Multiple RBs, as exemplified by the red arrowhead were also observed. The average particle area was skewed toward RBs (0.58 μm^2^), and several ABs, as defined by large area and unequal binary fission events were observed. While this analysis was performed with limited numbers of inclusions, we note there is a statistically significant difference in intra-inclusion particle size between Patients 1 and 2 (Supplementary Figure 2). The mixture of a few EBs, RBs, and ABs observed in Figures 4B–D are reminiscent of inclusion morphology observed *in vitro* when infected cells are exposed to IFNγ. They also differ strikingly from inclusions observed after penicillin exposure, which contain a single large RB (Skilton et al., 2009), or after adenosine exposure, the latter being largely empty with a few EBs (Pettengill et al., 2009).

**Figure 5:**
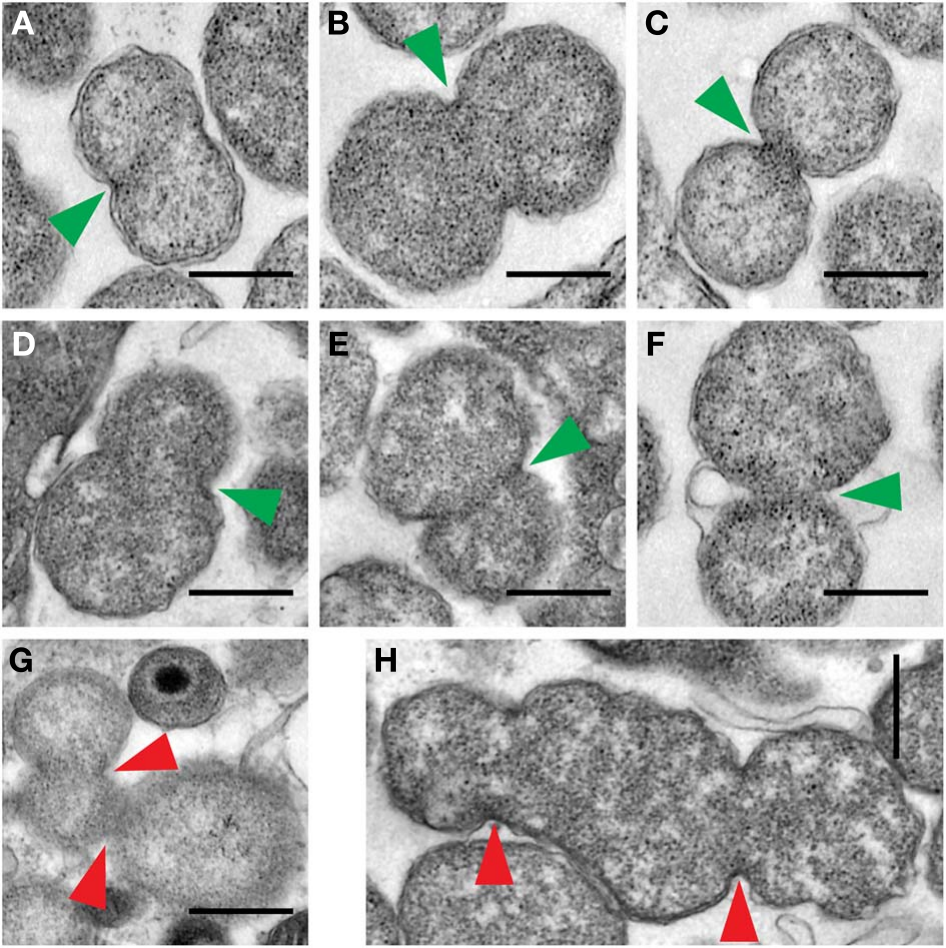
**Examples of RB binary fission detected in electron micrographs from Patient 2**. Three types of binary fission were observed, including, septum formation where the plane of division, indicated by a green arrowhead, is predicted to result in equal division **(A–C)**, or unequal division **(D–F)**, or where multiple septum formation events, indicated by the red arrowheads, were apparently present **(G–H)**. The scale bar indicates a distance of 0.5 μm.

Interestingly, particles with greater than EB-sized cross-sectional areas observed within inclusions from Patient 2 appear to display two types of binary fission characteristics (Figure 5). Some of them (Figures 5A–C) displayed roughly equal binary fission. However, several others (Figures 5D–F) displayed apparently unequal binary fission. Some fission events with apparently multi-septated ABs were also observed (Figures 5G,H). Because a decreased expression of chlamydial genes required for cytokinesis is observed during IFNγ -induced persistence *in vitro* (Byrne et al., 2001; Belland et al., 2003a), it is possible that the latter events may result from the effects of IFNγ.

### Parallel molecular analysis of cervical specimens with ultrastructural data identifies a patient with a bacterial profile consistent with persistent growth forms

A substantial body of indirect evidence supports the possibility that *C. trachomatis* adapts, and survives long-term, during human infection (Hogan et al., 2004; Brunham and Rey-Ladino, 2005; Wyrick, 2010). However, direct proof that the bacterium can enter into a persistent mode of growth in human infections requires morphologic evidence of aberrant forms to be substantiated by molecular evaluation of bacterial growth patterns to determine if the latter coincide with patterns observed *in vitro*. As our second study protocol was designed to harvest, and immediately preserve, parallel *ex vivo* endocervical samples for EM, nucleic acid, and infectious particle (IFU) analyses, we next examined multiple parameters of chlamydial growth in the 2 patients for whom we had ultrastructural inclusion data. First, we observed the two patients greatly contrasted in their IFU burden; Patient 1 had an IFU of greater than 336,168 and Patient 2 an IFU of 67 (Table 1). The median IFU in this patient population was 4802; thus, these two patients represent cases at the very highest, and at the lower end, of the spectrum of young women with NAAT and culture-positive infections. Second, using total nucleic acid derived from the same endocervical sample used to generate IFU values, we enumerated genome copy numbers. Importantly, both patients had high genome copy numbers despite the strikingly lower IFU counts in Patient 2 whose genome copy number actually exceeded that of Patient 1 (Table 1). These data indicate that, relative to Patient 1, a significantly smaller proportion of the total bacterial burden were infectious EB particles in Patient 2. Third, we evaluated the relative RNA expression levels of *euo*, an early-expressed chlamydial gene that codes for Euo (Zhang et al., 1998), and *omcB*, a gene expressed later in the developmental cycle that is repressed by Euo (Rosario and Tan, 2012). The ratio of *euo* to *omcB* mRNA expression also indicated contrasting patterns of growth in the two patients, with a significant predominance of *omcB* transcripts in Patient 1 and a significant predominance of *euo* transcripts in Patient 2 (Table 1). These data taken together with the morphological evidence, suggests that endocervical infection in both patients is characterized by a heavy bacterial genome burden, but, in contrast to the highly productive growth of *Chlamydia* in patient 1, the chlamydial growth profile of patient 2 is relatively asynchronous, and predominated by aberrant and early stage bacterial forms.

**Table 1:** **Measurement of multiple parameters of growth of *C. trachomatis* isolated from the cervix of two infected patients**.

**Growth parameter**	**Patient 1**	**Patient 2**
IFU	336,168	67
Genome copy number	359,002	895,313
Genome copy number: IFU	1.067924	13,362.88
*Euo: omcB*	1.865	10.0

*Shown are total cervical infectious forming units (IFU), genome copy numbers, ratio of IFU to genome copy number and ratio of Euo to omcB mRNA recovered from an endocervical swab for each patient*.

### IFNγ and indoles in secretions identify potential correlates for differing chlamydial growth patterns between patients

Next, we examined the infecting *ompA* genotype, clinical history, and key immune and environmental elements that might explain the contrasting growth patterns seen in Patient 1 and Patient 2. We determined that both patients were infected with an identical E *ompA* genotype (data not shown), neither had a documented history of *C. trachomatis* infection and both had a mucopurulent cervical discharge but no vaginal discharge. Both were negative for *N. gonorrhoea* and *T. vaginalis* but had a positive Nugent score (8 for Patient 1 and 7 for Patient 2, respectively) indicating BV. Finally, we assayed genital secretions to reveal the local concentration of indoles, and IFNγ. Indoles were detected in both patients, with the levels in Patient 1 being approximately twice as high as those in Patient 2 (278 vs. 159 mM). Importantly, while Patient 1 had a very low local concentration of IFNγ, this response was robust in Patient 2 (1.45 vs. 68.95 pg/mg protein, 35.48 vs. 613.09 pg/ml) (Aiyar et al., 2014).

## Discussion

In this study, we sought to develop techniques to identify the growth forms of *C. trachomatis* present in the human endocervix, the most common site of infection in women, with the concurrent evaluation of other biomarkers that could aid in classifying the spectrum of infections that occur at this site. During the course of this study, we established methodology to preserve bacteria and host epithelial cells for detailed morphologic and molecular analyses. This novel approach allowed us to document the presence of inclusions containing normal, morphologically aberrant, or a combination of these chlamydial forms, in epithelial cells. Importantly, in a pilot study, we could identify two patients with high numbers of bacterial genomes in their cervices, but, with contrasting bacterial growth patterns as revealed by parallel measurements of IFU, inclusion morphology, and transcriptional analyses of key early and late expressed chlamydial genes. Specifically, one patient (Patient 1) had a chlamydial growth pattern predominated by high numbers of infectious particles, whereas the other (Patient 2) displayed a very low IFU with inclusions that predominantly included both aberrant and normal RB forms. Coincident with these observations, a minimal IFNγ response was detected in Patient 1 secretions, in contrast to a more robust IFNγ response in Patient 2. A significant level of indoles was detected in both patients, likely consistent with being BV-positive.

Relatively few studies have described the morphology and abundance of chlamydial inclusions in the human cervix. In 1938, Braley examined cervical biopsies and conjunctival smears from respective mother-infant pairs in which the infant was diagnosed with inclusion blennorrhea (Braley, 1938). Inclusions were readily identified by Giemsa staining in infant conjunctival epithelial cells, but were only found in the cervix of a small proportion of women and were few in number (*ibid*). Three decades later, using EM on biopsied transition zone and adjacent cervix in two women co-infected with *C. trachomatis* and *N. gonorrhoeae*, Swanson identified inclusions in the transitional zone and in the columnar epithelium (Swanson et al., 1975). Of note, only classical inclusions were investigated and described, and antigen-specific staining procedures were not undertaken. Subsequent endocervical infection studies predominantly focused on diagnostics (presence/absence of EB). Exceptions to this were a study by Dunlop et al. who report observing chlamydiae in 7/159 patients by EM and present a micrograph of a single normal inclusion (Dunlop et al., 1989), and a study by Bragina et al., who reported the presence of small, aberrant bacterial forms in a cervical smear of a patient after inappropriate antibiotic treatment (Bragina et al., 2001). *In vivo* identification of human infections with persistent *C. trachomatis* forms is clinically important as these could: (1) provide a reservoir of persistent bacteria that reactivate when conditions in the local micro-environment are permissive; (2) evade key pathways involved in immune clearance; (3) contribute to the chronic inflammatory process; (4) underlie the recalcitrance of chlamydiae to clearance by certain antibiotics; and (5) avoid elimination by vaccine-induced immunity optimized to eradicate replicating forms.

In the study described here, we were able to demonstrate the utility of cytobrushes to sample intact infected cells from endocervix, finding similar numbers of inclusions to those observed in adult conjunctival smears (Braley, 1938). This methodology is important as cytobrushes (i) are non-invasive, unlike biopsies that are extremely difficult to obtain at this site, (ii) provide a method for longitudinally sampling, and (iii) can be immediately placed in a medium of choice for *ex vivo* analysis. Thus, by immediate immersion of cytobrushes into a modified EM buffer, we could preserve bacterial growth forms *ex vivo* for analyses by widefield or EM. The subsequent EM studies provide the first collection of micrographs of multiple *in vivo* inclusions. In one patient examined, inclusions were relatively homogeneous, predominantly contained EBs, and are therefore late in the developmental cycle. In contrast, in the second patient, we identified inclusions with predominantly RBs, predominantly ABs, a mix of RB and aberrant forms, a heterogeneous mix of mid-stage forms, and a classical late stage inclusion containing EBs. This wide spectrum of inclusion morphology suggests the possibility of different microenvironments in the same tissue in which factor/s driving bacterial development or persistence could be spatially and/or temporally variable.

The IFNγ-mediated host response to *C. trachomatis* infection has been studied in depth. In human cells, IFNγ induces the tryptophan-catabolizing enzyme IDO1 that catabolizes tryptophan to kynurenine (Shemer and Sarov, 1985; Byrne et al., 1986, 1989; Carlin et al., 1989; Beatty et al., 1994a; Brunham and Rey-Ladino, 2005). This depletion interferes with the growth of *C. trachomatis*, which is a tryptophan auxotroph (*ibid*). At sufficient and sustained concentrations of IFNγ *in vitro, C. trachomatis* can be eliminated from human host cells establishing this cytokine as a key component in local host defense (Byrne et al., 1989). In tryptophan-limiting but sub-inhibitory concentrations of IFNγ *in vitro* however, a scenario likely often encountered *in vivo*, chlamydiae enter into the persistent mode of growth (Beatty et al., 1993, 1994a). Transcriptome analysis of this altered state reveals a gene expression profile consistent with continued expression of genes governing DNA replication but not with those genes involved in bacterial cell division (Belland et al., 2003a). This includes upregulation of genes involved in tryptophan utilization, DNA repair and recombination, phospholipid biosynthesis and translation (*ibid*). A number of early genes are also upregulated, and in particular, *euo* (30-fold increase), which encodes a DNA-binding protein that binds to a late gene promoter region and is the transcriptional regulator of *omcB* (Zhang et al., 1998, 2000; Rosario and Tan, 2012). Down-regulation of genes involved in RB to EB differentiation (such as *omc*AB), proteolysis, peptide transport, and cell division are also noted (Belland et al., 2003a). Removal of IFNγ leads to a rapid reactivation with gene expression rapidly returning to control levels, for example, *euo* expression drops 20-fold in 12 h (*ibid*). The knowledge that *euo* and *omcB* are differentially expressed in active and IFNγ-driven persistent growth, the commonality of this expression pattern to other persistence inducers, their lack of co-expression and the known stability of both mRNAs, all suggested the utility of choosing *euo/omcB* expression ratio as a putative biomarker of *in vivo* growth. By calculating this ratio, we were able to corroborate morphological, IFU, and genome data that the two patients described in the study had contrasting patterns of chlamydial growth. Future studies should confirm the utility of this combination of biomarkers to detect and determine the consequences of a spectrum of *C. trachomatis* infection seen in the human endocervix. Transcriptional analyses of a wider panel of genes should also reveal the common inducers of persistence in the genital tract. IFNγ is likely to be one of these stressors, and our laboratory and others have identified a clear association with, but spectrum of, IFNγ concentration in genital secretions during *C. trachomatis* infection that wanes after resolution of infection (Ficarra et al., 2008; Aiyar et al., 2014). Certainly the transcriptional changes that occur in the presence of IFNγ and result in persistent growth appear to constitute a persistence stimulon and suggest a coordinated biological response that has evolved to allow the organism to rapidly respond to, and survive, immunological pressure by a period of resistance followed by a rapid recovery after waning of the host response or by supplementation of tryptophan (Belland et al., 2003a). Importantly, by expressing tryptophan synthase during tryptophan starvation genital *C. trachomatis* serovars can uniquely salvage indole to supplement tryptophan, and indole-producing organisms such as bacteria associated with BV and *T. vaginalis* have been suggested to be the enabling co-factors (Caldwell et al., 2003; Morrison, 2003; Aiyar et al., 2014). In support of this, both patients in this pilot study were diagnosed with BV and indoles were present in their vaginal secretions. We speculate that the weak IFNγ response noted in Patient 1 may have been further compromised by indoles. In contrast, the lower levels of indoles detected in Patient 2 may be insufficient to overcome the restriction imposed by a robust IFNγ response.

While undoubtedly challenging, human studies targeted at elucidating if, when and how *C. trachomatis* enters into a persistent growth mode *in vivo* has been repeatedly stressed as *a priori* since these will likely significantly deepen our knowledge of the pathogenesis of this disease and hence stimulate new targeted treatment and prevention strategies (Wyrick, 2010; Schoborg, 2011). Because BV and *T. vaginalis* co-infections are so prevalent in *C. trachomatis* infected women (Koumans et al., 2007; Sutton et al., 2007), the role they play in supplementing local tryptophan, compromising IFNγ-mediated immunity and aiding establishment of chronic infections is also critical (Caldwell et al., 2003; Aiyar et al., 2014). The approaches described here could be applied to evaluate chlamydial infections at other sites *in vivo*. Further, as the commitment to a vaccine grows, we must also consider the multiple implications of our, and other recent, findings. Importantly, if the community is unable to develop a sterilizing vaccine, then we will likely need to formulate a vaccine to include antigens that are predominantly expressed by persistent growth forms. In addition we will need to determine the type of, and local level, of immunity and environmental milieu that will be most effective at eradicating, and least likely to drive bacteria into, a persistent growth mode, or to cause tissue damage. We believe these studies, and the potential biomarker panel we have investigated, form a strong platform for beginning to answer these questions. They also provide an avenue to explore the effect, and consequences of genital co-infections *on C. trachomatis* survival *in vivo*.

### Conflict of interest statement

The authors declare that the research was conducted in the absence of any commercial or financial relationships that could be construed as a potential conflict of interest.

## Supplementary material

Supplementary Figure 1 **Ultrastructural image of an inclusion from Patient 2**. The entire inclusion cross-sectional area was not obtained for this inclusion and therefore quantitative measurements were not performed. EB morphotypes predominated in this inclusion.

Supplementary Figure 2 **Upper Panel: Particle cross-sectional area (Y-axis) for every particle detected in the two inclusions from Patient-1 (Figure 3 in the manuscript)**. Particle identity was assigned in numerical sequence by Fiji (X-axis). Particle cross-sectional area was calculated as described in the Materials and Methods section. The light green shading indicates the anticipated area for EBs, while the anticipated area for RBs is shaded in light blue. Lower Panel: Particle cross-sectional area for particles from the four inclusions from Patient-2 shown in Figure 4 of the manuscript. The average cross-sectional area for particles from Patient-1 was 0.126 ± 0.109 μm^2^, while the average cross-sectional area for particles from Patient-2 was 0.431 ± 0.373 μm^2^. The difference in particle cross-sectional areas within inclusions from Patient-1 and Patient-2 was determined to be statistically significant by the Wilcoxon rank-sum test: P(two-sided) = 10e-16.
