# Supplementary figures and images for: Morphologic and molecular evaluation of Chlamydia trachomatis growth in human endocervix reveals distinct growth patterns
Source: Front Cell Infect Microbiol. 2014 Jun 10;4:71. doi: 10.3389/fcimb.2014.00071 (PMC4050528; doi:10.3389/fcimb.2014.00071)

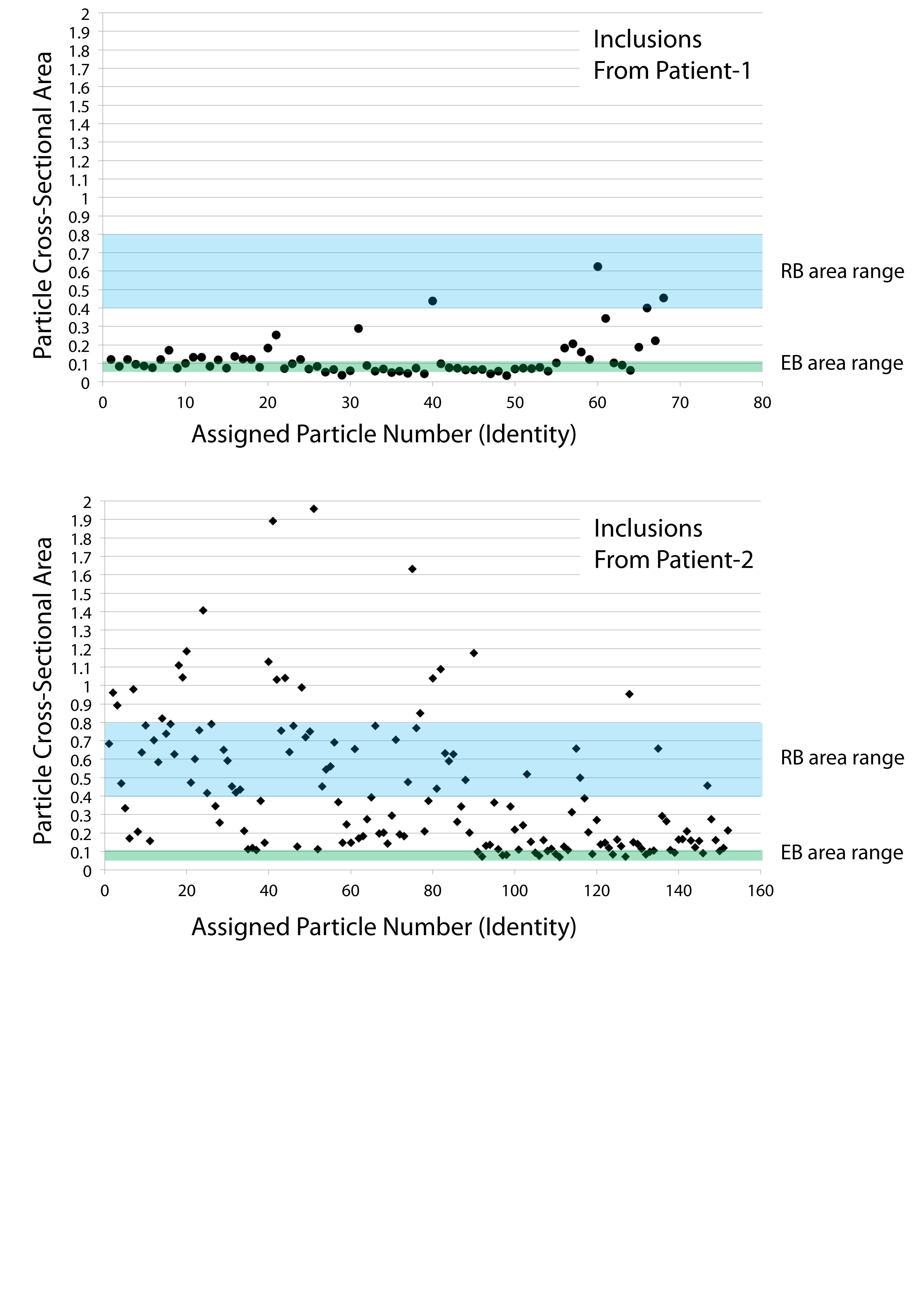

Supplement: Supplementary Figure 1 — Ultrastructural image of an inclusion from Patient 2. The entire inclusion cross-sectional area was not obtained for this inclusion and therefore quantitative measurements were not performed. EB morphotypes predominated in this inclusion. [file Presentation1.ZIP › 91720_Supp Fig 2.TIF]

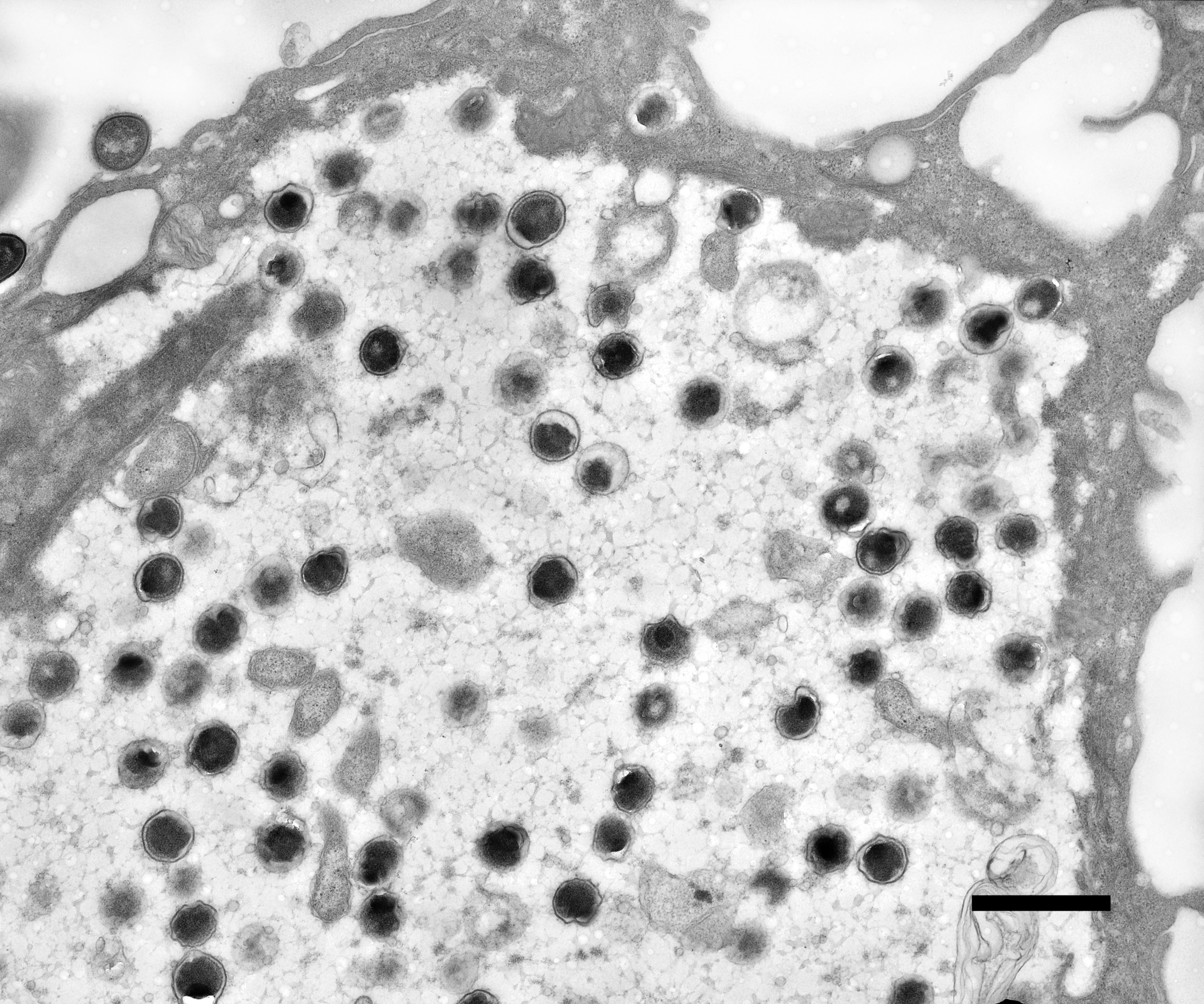

Supplement: Supplementary Figure 1 — Ultrastructural image of an inclusion from Patient 2. The entire inclusion cross-sectional area was not obtained for this inclusion and therefore quantitative measurements were not performed. EB morphotypes predominated in this inclusion. [file Presentation1.ZIP › 91720_Supp Fig 1.TIF]
